# Parsing clinical text: how good are the state-of-the-art parsers?

**DOI:** 10.1186/1472-6947-15-S1-S2

**Published:** 2015-05-20

**Authors:** Min Jiang, Yang Huang, Jung-wei Fan, Buzhou Tang, Josh Denny, Hua Xu

**Affiliations:** 1 The University of Texas School of Biomedical Informatics at Houston, Houston, TX, USA; 2 Kaiser Permanente, San Diego, CA, USA; 3 Shenzhen Graduate School of Harbin institute of Technology, Shenzhen, China; 4 Department of Medicine, Vanderbilt University, School of Medicine Nashville, TN, USA; 5 Department of Biomedical Informatics, Vanderbilt University, School of Medicine, Nashville, TN, USA

**Keywords:** Medical language processing, natural language processing, parsing, clinical text, NLP

## Abstract

**Background:**

Parsing, which generates a syntactic structure of a sentence (a parse tree), is a critical component of natural language processing (NLP) research in any domain including medicine. Although parsers developed in the general English domain, such as the Stanford parser, have been applied to clinical text, there are no formal evaluations and comparisons of their performance in the medical domain.

**Methods:**

In this study, we investigated the performance of three state-of-the-art parsers: the Stanford parser, the Bikel parser, and the Charniak parser, using following two datasets: (1) A Treebank containing 1,100 sentences that were randomly selected from progress notes used in the 2010 i2b2 NLP challenge and manually annotated according to a Penn Treebank based guideline; and (2) the MiPACQ Treebank, which is developed based on pathology notes and clinical notes, containing 13,091 sentences. We conducted three experiments on both datasets. First, we measured the performance of the three state-of-the-art parsers on the clinical Treebanks with their default settings. Then we re-trained the parsers using the clinical Treebanks and evaluated their performance using the 10-fold cross validation method. Finally we re-trained the parsers by combining the clinical Treebanks with the Penn Treebank.

**Results:**

Our results showed that the original parsers achieved lower performance in clinical text (Bracketing F-measure in the range of 66.6%-70.3%) compared to general English text. After retraining on the clinical Treebank, all parsers achieved better performance, with the best performance from the Stanford parser that reached the highest Bracketing F-measure of 73.68% on progress notes and 83.72% on the MiPACQ corpus using 10-fold cross validation. When the combined clinical Treebanks and Penn Treebank was used, of the three parsers, the Charniak parser achieved the highest Bracketing F-measure of 73.53% on progress notes and the Stanford parser reached the highest F-measure of 84.15% on the MiPACQ corpus.

**Conclusions:**

Our study demonstrates that re-training using clinical Treebanks is critical for improving general English parsers' performance on clinical text, and combining clinical and open domain corpora might achieve optimal performance for parsing clinical text.

## Introduction

Parsing is the process of assigning syntactic structures to input strings according to grammar. Early studies often relied on symbolic parsing approaches that used manually created deterministic grammars to generate parse trees. With the increased availability of annotated corpora in the 1990's, such as the English Penn Treebank Wall Street Journal corpus[1], statistical approaches, which identify the best parse tree based on probabilities learned from the annotated Treebank, have been widely used in syntactic parsing and have shown great performance [2-4]. For example, many statistical parsers have been developed based on the Penn Treebank [1]. In 1995, Magerman [2] developed one of the first parsers that showed that high-performance parsing could be achieved using only the Treebank based corpus. In his approach, he used the decision-tree learning technique to construct a parse tree of every sentence and evaluation on the Peen Treebank showed an F-measure of 84.7%. In 1999, Collins [3] demonstrated the use of generative models in syntactic parsing. He extended his probabilistic parser developed in 1996 with three generative models to calculate all the probabilities of the parse tree head nodes including adjunct/complement distinction and wh-movement. Evaluation showed that these models surpassed Megerman's and his previous parsers and achieved a F-measure of 87.8%. In 2004, Bikel [5] used an Expectation-Maximization Model to estimate some feature space parameters in the Collins model. The Bikel parser improved the performance of the Collins' parser and achieved a better F-measure for all the parameters that it tested, demonstrating that the Bikel parser was a robust and reliable emulation of the Collins parser. Charniak and Johnson [6] presented a discriminative re-ranking method for constructing high-performance statistical parsers. Based on a coarse-to-fine generative parser, they constructed sets of 50-best parse trees and used them as input into a Maximum Entropy re-ranker, which then selected the best parse. Their parser outperformed all the previous generative models and achieved an F-measure of 91.0%. More recently, McClosky et al. [7] presented a two-phase parser that consisted of the Charniak parser and a bootstrapping method for self-training on raw sentences. The McClosky parser boosted the performance of the one-phase Charniak parser by 0.8% (F-measure). Besides the above mentioned lexicalized parsers, the Stanford parser [4], which was initially developed based on un-lexicalized PCFG (probabilistic context-free grammar), has also shown great performance and has been widely used in different domains. These state-of-the-art parsers have also been applied to the biological domain to process biomedical literature. For example, Lease and Charniak [8] extended the Charniak parser to process the GENIA corpus [9] generated from MEDLINE abstracts by leveraging existing domain-specific lexical resources to augment training with the Penn Treebank. More recently, Clegg and Shepherd [10] developed an evaluation method by using dependency graphs as an intermediate representation wherein they compared four parsers: the Collins parser [3], the Bikel parser [5], the Stanford parser [4], and the Charniak-Lease parser [6], on the GENIA corpus. Their results showed that the Bikel and Charniak-Lease parsers achieved better performance than the others; but the overall performance of all the parsers dropped when compared with results from the Penn Treebank.

Over the past two decades, there is a growing interest in developing high performance NLP systems for the medical domain. Much of the detailed patient information in the patient records is embedded in narrative clinical notes and NLP provides a means to unlock this information to facilitate its utilization in other computerized clinical applications. Many clinical NLP systems have been developed [11-20] and have shown great potential in various clinical applications [21]. Despite the success of existing clinical NLP systems on information extraction tasks, few of them have implemented full syntactic parsing functionality. Even though clinical text is known for its more restricted semantic constraints [22], obtaining accurate and deep syntactic structures of clinical sentences is appealing for building high-performance clinical NLP systems. The lack of research in syntactic parsing of clinical text could be due to the telegraphic style of clinical notes (e.g., many abbreviations and frequent ungrammatical sentences), rendering them intractable for syntactic parsing. Some previous studies extended the general English parsers such as the Stanford Parser using medical lexicon for clinical text processing [23], but no formal evaluation of parsing has been done for these parsers. Fortunately, recent initiatives in the clinical NLP community have led to generation of annotation guidelines, as well as annotated corpora for parsing clinical text. Fan et al. extended the Penn Treebank annotation guidelines to handle ill-formed clinical sentences and created a Treebank of 25 progress notes from University of Pittsburgh Medical Center (UPMC) [24]. Another newly annotated clinical corpus, named MiPACQ, was created using pathology and other clinical notes from the Mayo Clinic. MiPACQ contains multiple layers of annotations, including named entities, syntactic parse trees, dependency parse trees, and semantic role labeling on 13,091 sentences [25]. Therefore, it is timely to explore the performance of existing statistical parsers and develop new parsing strategies for clinical text.

In this study, we evaluated the performance of three state-of-the-art parsers: the Stanford parser [4], the Bikel parser [5] and the Charniak parser [6], using two clinical Treebanks including the Treebank of progress notes reported in Fan et al. [24] and the MiPACQ Treebank. The purposes of this study were three-fold: (1) to evaluate the default performance of existing state-of-the-art English parsers on clinical text; (2) to assess the value of clinical Treebanks for re-training of existing general English parsers; and (3) to investigate whether combining the Penn Treebank and the clinical Treebanks can improve the performance of parsers on clinical text. To the best of our knowledge, this is the first comprehensive study that has investigated syntactic parsing of clinical text using multiple state-of-the-art parsers and Treebanks from both the general English domain and the clinical domain.

## Methods

### The clinical Treebank

In this study, we used three Treebanks: 1) the ProgressNotes Treebank built in Fan et al. [24] 2) the MiPACQ Treebank described in Albright et al. [25] and 3) the "WSJ (The Wall Street Journal)" Treebank, which contains two sections of the Penn Treebank that was purchased from the Linguistic Data Consortium. In both the annotated clinical corpora, we found the existence of some very short fragments in the notes which is not desirable for full parsing. For example, some fragments only included the name of section headers in clinical notes. Therefore, we precluded the annotation of sentences with less than 5 tokens from both the clinical Treebanks in our studies. After the filtering, we had 1025 sentences in the progress notes Treebank and 10661 sentences in the MiPACQ Treebank. Table 1 shows the details of the three Treebanks used in our study.

**Table 1:** Information about the three Treebanks used in this study.

Corpus Description	Description	# of sentences
ProgressNotes	25 progress notes from UPMC	1025

MiPACQ	Pathology and other clinical notes from Mayo Clinic	10661

WSJ	Two sections of the WSJ corpus	3914

### The parsing experiments

Initially, we planned to follow Clegg and Shepherd's study [10], which compared four parsers. We noticed that the package of the Collins parser did not include a simple way to re-train the parser using a different corpus, therefore we excluded the Collins parser. As a result, we used three parsers in this study: the Stanford parser [4], the Bikel parser [5] and the Charniak parser [6]. For the Stanford parser, the lexicalized version was used. Sentences with manually annotated POS tags were then supplied to each parser to generate parse trees. Three experiments were conducted for each parser as described below:

1) Evaluate performance of parsers with their default settings: In this experiment, we directly applied the three parsers to process all POS-tagged sentences for both the Treebanks. All the parsers were invoked with their default settings and models, which had been trained on the Penn Treebank. The Parse trees generated by each parser were then compared with the gold standard Treebank and the performance of each parser was reported (please see the Evaluation section).

2) Re-train parsers on the clinical Treebank: To assess if retraining on the clinical corpus could improve the parsers' performance in each corpus, we conducted 10-fold cross validation evaluation for each parser. The cross-validation involved dividing the clinical corpus equally into 10 parts, and training the parser on 9 parts with testing on the remaining part each time. We repeated the same procedure 10 times, one for each part, and then combined the results from the 10 parts to report the performance.

3) Combine the Penn Treebank and the clinical Treebank: The most obvious method to make use of the Penn Treebank is to directly combine the Penn Treebank and clinical Treebanks as the training corpus. Due to the large size of the Penn Treebank, in this experiment, we used only the first two sections of the WSJ corpus in the Penn Treebank (3914 sentences in total). We used the 10-fold cross validation evaluation as explained in section 2) above. However, for the training set, we combined the WSJ corpus with 9 parts from the clinical corpus.

### Evaluation

As described previously, for each parser, we conducted above three experiments and implemented 10 fold cross validation. For each testing sentence, a parse tree generated by the parser was compared with the corresponding gold standard in the Treebank and evaluated using the PARSEVAL EVALB package (http://nlp.cs.nyu.edu/evalb/), which is commonly used for evaluating parsers. For each sentence, the following PARSEVAL measures were calculated:

**Bracketing Recall (BR) **= (The number of Correct Constituents in the Systems' Parse Tree) / (The Number of Constituents in the Gold Standard Parse Tree)

**Bracketing Precision (BP) **= (The number of Correct Constituents in the Systems' Parse Tree) / (The Number of All Constituents in the System's Parse Tree)

**Bracketing F-measure (BF) **= 2xBPxBR/(BP+BR)

The average BR, BP, and BF across both Treebanks were reported as the final results.

## Results

Table 2 shows the experimental results on the ProgressNotes Treebank. The Stanford parser achieved the best performance of 70.30% BF, with the default settings. Compared to the default setting, re-training on the clinical Treebank improved the performance for all the three parsers, and the biggest boost was achieved by the Bikel parser (from a F-score of 66.60% to 72.45%). When the combined corpora of both progress notes and WSJ articles were used for training, the BF of the Charniak parser increased from 70.01% (only progress notes used) to 73.53%; however, both the Stanford and the Bikel parsers slightly dropped their performance.

**Table 2:** Results on 1025 sentences from progress notes.

Parser	Corpus	BR (%)	BP (%)	BF (%)
Stanford	WSJ	70.32	70.27	70.30
	
	ProgressNotes	76.22	71.31	73.68
	
	WSJ + ProgressNotes	74.27	71.16	72.68

Bikel	WSJ	64.20	69.20	66.60
	
	ProgressNotes	71.85	73.05	72.45
	
	WSJ + ProgressNotes	70.85	73.92	72.35

Charniak	WSJ	62.91	75.03	68.44
	
	ProgressNotes	65.82	74.78	70.01
	
	WSJ + ProgressNotes	75.89	71.31	73.53

Table 3 shows the results obtained using the MiPACQ Treebank. With the default setting, the Stanford parser again achieved the best performance among all the parsers. Upon re-training on the MiPACQ Treebank alone, all the three parsers had a big leap in performance with the Charniak parser showing the maximum increase (increased from a BF of 74.18% to 83.54%). Re-training on the combined Treebanks of MiPACQ and WSJ led to marginal increases in performance for all the parsers. Among all the parsers, the Stanford parser achieved the best BF of 84.15% when both MiPACQ and WSJ Treebanks were used.

**Table 3:** Results on 10661 sentences from MiPACQ corpus.

Parser	Corpus	BR (%)	BP (%)	BF (%)
Stanford	WSJ	75.54	74.41	74.97
	
	MiPACQ	84.28	83.16	83.72
	
	WSJ + MiPACQ	84.30	84.00	84.15

Bikel	WSJ	73.49	75.78	74.62
	
	MiPACQ	77.59	78.09	77.84
	
	WSJ + MiPACQ	77.43	78.63	78.03

Charniak	WSJ	70.63	78.11	74.18
	
	MiPACQ	80.88	86.39	83.54
	
	WSJ + MiPACQ	80.65	86.76	83.59

## Discussion

Full syntactic parsing is an important area of clinical NLP research, but it has not been extensively explored so far. In this study, we conducted the first formal evaluation to compare the performance of three state-of-the-art English parsers on clinical notes using two clinical Treebanks. When both clinical and WSJ corpora were combined to train the parsers, the highest average BFs of 84.15% and 73.53% were achieved by the Stanford parser for the MiPACQ corpus and the Charniak parser for the ProgressNotes corpus respectively.

As expected, existing parsers achieved lower performance on clinical text than previously reported results on general English text, when they were directly applied to clinical text. For instance, on the MiPACQ corpus, the Stanford parser showed a decrease of 11.35% in BF (from 86.32% in [8] to 74.97% in this study). When the existing parsers were re-trained on the clinical Treebanks, their performance increased. For the progress notes Treebank, there were 3.38%, 5.85% and 1.57% increases in BF for the Stanford, Bikel and Charniak parser respectively. For the MiPACQ corpus, the increases were 8.19%, 3.22% and 9.36%, which were much higher than increases in progress notes corpus, probably due to the larger sample size of the MiPACQ corpus (about 10 times larger than the progress notes corpus - 10,661 vs.1,025 sentences). These findings suggest that re-training on clinical corpora is necessary for developing high-performance statistics-based parsers for clinical text. It also indicates the need for building annotated clinical Treebanks.

Although there is growing interest in building annotated clinical corpora, the sizes of these corpora are often limited due to the high cost of physician annotators. Large-scale corpora from other domains, such as the Penn Treebank, are available and should be leveraged for clinical parsing. That is the motivation of the combination approach proposed in this study. For progress notes, direct combination of the WSJ corpus and the clinical corpus showed varying results among the three parsers. It largely improved the performance of the Charniak parser; but reduced the performance of the Stanford and the Bikel parsers. The inconsistency may be due to the small sample size of the ProgressNotes Treebank itself. For the MiPACQ corpus, which is 10 times larger than the ProgressNotes corpus, direct combination of WSJ and clinical corpora marginally but consistently improved the performance for all the three parsers (increases of BF ranging from 0.05% - 0.43%). These results suggest that it is possible to leverage existing corpora in the open domain to improve parsing of clinical text. However, instead of simply combining different corpora, sophisticated methods, such as domain adaptation techniques [26-28], should be investigated to improve parsing in the medical domain. Furthermore, we are also interested in semi-supervised learning methods such as co-training, which may help build large-scale clinical corpus from unlabeled data.

When existing parsers were directly applied to clinical text, a main category of errors was the failure to recognize structures of clinical sentences, which are often ill-formed. We also analyzed errors from parsers re-trained on clinical corpus and categorized them into the following major groups:

1) Ambiguity of coordination: For example, in the sentence "CXR was repeated and found to have no signs of infiltrate and scant pulmonary congestion", "infiltrate" and "scant pulmonary congestion" should be both linked to "no signs". But the parser recognized it as two phrases: "no signs of infiltrate" and "scant pulmonary congestion", which was wrong.

2) Ambiguity of prepositional phrase (PP) attachment: For example, in the sentence "He denies any problem with chest pain, dyspnea on exertion at this time", the parser did not identify the prepositional phrase 'on exertion' as a modifier to 'dyspnea'. Clinical knowledge will be useful for solving this type of ambiguity.

3) Errors in the non-terminal symbol 'NX': NX was used to mark the head noun within a complicated noun phrase in the annotation guideline. However, parsers had trouble identifying them correctly.

Our study has the following limitations. The development of the ProgressNotes Treebank was based on pre-processed parsed trees from the Stanford parser [24]. Although annotators have carefully reviewed all the parsed trees, bias could have been introduced into the gold standard and thus, may result in favorable performance for the Stanford parser. In this study, only certain types of clinical notes are involved. In the future, we plan to extend this study to other types of clinical notes such as discharge summaries, to assess the generalizability of our findings. In addition, not all state-of-the-art parsers were included in this study. We plan to include more parsers in the next study, e.g., the Berkeley parser developed by Petrov and Klein [29].

## Conclusions

We conducted a formal evaluation to investigate the use of three state-of-the-art parsers in the medical domain. Our results showed that the Stanford parser achieved the best performance when they were directly applied to the clinical text. Moreover, retraining on the annotated clinical corpus significantly improved all parsers' performance, indicating the need to create large clinical Treebanks. In addition, we demonstrated that combining open domain corpora such as the Penn Treebank with clinical corpora could further improve the performance of parsers on clinical text. Therefore, more sophisticated methods for combining corpora that can leverage annotated corpora from outside domains for clinical parsing would be worth investigating.

## Competing interests

The authors declare that they have no competing interests.

## Authors' contributions

The work presented here was carried out in collaboration between all authors. MJ, HX, BT designed methods and experiments. MJ carried out the experiments. MJ, YH and JF, HX were involved in the development of corpus, MJ, HX, JD interpreted the results and wrote the paper. All authors have attributed to, seen and approved the manuscript.

## Declaration

Publication costs for the article were sourced from NLM grant 2R01LM010681-05.

This article has been published as part of *BMC Medical Informatics and Decision Making *Volume 15 Supplement 1, 2015: Proceedings of the ACM Eighth International Workshop on Data and Text Mining in Biomedical Informatics. The full contents of the supplement are available online at http://www.biomedcentral.com/bmcmedinformdecismak/supplements/15/S1.

## References

[B1] Marcus MP Marcinkiewicz MA Santorini B Building a large annotated corpus of English: the penn treebank Comput Linguist 1993 19 2 313 330

[B2] Magerman DM Statistical decision-tree models for parsing Proceedings of the 33rd annual meeting on Association for Computational Linguistics 1995 Cambridge, Massachusetts: Association for Computational Linguistics

[B3] Collins M Three generative, lexicalised models for statistical parsing Proceedings of the 35th Annual Meeting of the Association for Computational Linguistics and Eighth Conference of the European Chapter of the Association for Computational Linguistics 1997 Madrid, Spain: Association for Computational Linguistics

[B4] Klein D Manning CD Accurate unlexicalized parsing Proceedings of the 41st Annual Meeting on Association for Computational Linguistics - Volume 1 2003 Sapporo, Japan: Association for Computational Linguistics

[B5] Bikel DM On the parameter space of generative lexicalized statistical parsing models 2004

[B6] Charniak E Johnson M Coarse-to-fine n-best parsing and MaxEnt discriminative reranking Proceedings of the 43rd Annual Meeting on Association for Computational Linguistics 2005 Ann Arbor, Michigan: Association for Computational Linguistics

[B7] McClosky D Charniak E Johnson M Effective self-training for parsing Proceedings of the main conference on Human Language Technology Conference of the North American Chapter of the Association of Computational Linguistics 2006 New York, New York: Association for Computational Linguistics

[B8] Lease M Charniak E Parsing Biomedical Literaturethe Second International Joint Conference on Natural Language Processing (IJCNLP'05) 2005 2005 Jeju Island, Korea

[B9] Tateisi Y Ohta T dong Kim J Hong H Jian S Tsujii J The genia corpus: Medline abstracts annotated with linguistic information Third meeting of SIG on Text Mining, Intelligent Systems for Molecular Biology (ISMB) 2003

[B10] Clegg AB Shepherd AJ Benchmarking natural-language parsers for biological applications using dependency graphs BMC bioinformatics 2007 8 1 24 10.1186/1471-2105-8-24 17254351 PMC1797812

[B11] Mutalik S Chetana M Sulochana B Devi PU Udupa N Effect of Dianex, a herbal formulation on experimentally induced diabetes mellitus Phytother Res 2005 19 5 409 415 10.1002/ptr.1570 16106394

[B12] Sager N Friedman C Lyman M Medical language processing: computer management of narrative data 1987 Reading, MA: Addison-Wesley

[B13] Friedman C Alderson PO Austin JH Cimino JJ Johnson SB A general natural-language text processor for clinical radiology J Am Med Inform Assoc 1994 1 2 161 174 10.1136/jamia.1994.95236146 7719797 PMC116194

[B14] Hripcsak G Austin JH Alderson PO Friedman C Use of natural language processing to translate clinical information from a database of 889,921 chest radiographic reports Radiology 2002 224 1 157 163 10.1148/radiol.2241011118 12091676

[B15] Haug PJ Koehler S Lau LM Wang P Rocha R Huff SM Experience with a mixed semantic/syntactic parser Proc Annu Symp Comput Appl Med Care 1995 284 288 8563286 PMC2579100

[B16] Haug PJ Christensen L Gundersen M Clemons B Koehler S Bauer K A natural language parsing system for encoding admitting diagnoses Proc AMIA Annu Fall Symp 1997 814 818 9357738 PMC2233343

[B17] Aronson AR Effective mapping of biomedical text to the UMLS Metathesaurus: the MetaMap program Proc AMIA Symp 2001 17 21 11825149 PMC2243666

[B18] Savova GK Masanz JJ Ogren PV Zheng J Sohn S Kipper-Schuler KC Chute CG Mayo clinical Text Analysis and Knowledge Extraction System (cTAKES): architecture, component evaluation and applications J Am Med Inform Assoc 2010 17 5 507 513 10.1136/jamia.2009.001560 20819853 PMC2995668

[B19] Denny JC Smithers JD Miller RA Spickard A "Understanding" medical school curriculum content using KnowledgeMap Journal of the American Medical Informatics Association 2003 10 4 351 362 10.1197/jamia.M1176 12668688 PMC181986

[B20] Xu H Stenner SP Doan S Johnson KB Waitman LR Denny JC MedEx: a medication information extraction system for clinical narratives J Am Med Inform Assoc 2010 17 1 19 24 10.1197/jamia.M3378 20064797 PMC2995636

[B21] Meystre SM Savova GK Kipper-Schuler KC Hurdle JF Extracting information from textual documents in the electronic health record: a review of recent research Yearb Med Inform 2008 128 144 18660887

[B22] Friedman C Kra P Rzhetsky A Two biomedical sublanguages: a description based on the theories of Zellig Harris J Biomed Inform 2002 35 4 222 235 10.1016/S1532-0464(03)00012-1 12755517

[B23] Huang Y Lowe HJ Klein D Cucina RJ Improved identification of noun phrases in clinical radiology reports using a high-performance statistical natural language parser augmented with the UMLS specialist lexicon J Am Med Inform Assoc 2005 12 3 275 285 10.1197/jamia.M1695 15684131 PMC1090458

[B24] Fan J-w Yang EW Jiang M Prasad R Loomis RM Zisook DS Denny JC Xu H Huang Y Syntactic parsing of clinical text: guideline and corpus development with handling ill-formed sentences Journal of the American Medical Informatics Association 2013 20 6 1168 1177 10.1136/amiajnl-2013-001810 23907286 PMC3822122

[B25] Albright D Lanfranchi A Fredriksen A Styler WF Warner C Hwang JD Choi JD Dligach D Nielsen RD Martin J Towards comprehensive syntactic and semantic annotations of the clinical narrative Journal of the American Medical Informatics Association 2013 10.1136/amiajnl-2012-001317 PMC3756257 23355458

[B26] Mcclosky D Any domain parsing: automatic domain adaptation for natural language parsing 2010 Brown University

[B27] Roark B Bacchiani M Supervised and unsupervised PCFG adaptation to novel domains Proceedings of the 2003 Conference of the North American Chapter of the Association for Computational Linguistics on Human Language Technology - Volume 1 2003 Edmonton, Canada: Association for Computational Linguistics 126 133

[B28] McClosky D Charniak E Johnson M Reranking and self-training for parser adaptation Proceedings of the 21st International Conference on Computational Linguistics and the 44th annual meeting of the Association for Computational Linguistics 2006 Sydney, Australia: Association for Computational Linguistics 337 344

[B29] Petrov S Klein D Improved Inference for Unlexicalized Parsing 2007

